# Facile Synthesis of 4,4′-biphenyl Dicarboxylic Acid-Based Nickel Metal Organic Frameworks with a Tunable Pore Size towards High-Performance Supercapacitors

**DOI:** 10.3390/nano12122062

**Published:** 2022-06-15

**Authors:** Wenlei Zhang, Hongwei Yin, Zhichao Yu, Xiaoxia Jia, Jianguo Liang, Gang Li, Yan Li, Kaiying Wang

**Affiliations:** 1 Institute of Energy Innovation, College of Materials Science and Engineering & College of Information and Computer, Taiyuan University of Technology, Taiyuan 030024, China; zhangwenlei@tyut.edu.cn (W.Z.); yinhongwei@tyut.edu.cn (H.Y.); yuzhichao@tyut.edu.cn (Z.Y.); jiaxiaoxia@tyut.edu.cn (X.J.); 2 College of Mechanical and Vehicle Engineering, Taiyuan University of Technology, Taiyuan 030024, China; liangjianguo20@tyut.edu.cn; 3 College of Physics and Information Engineering, Minnan Normal University, Zhangzhou 361000, China; liyan_nmsd@163.com; 4 Department of Microsystems-IMS, University of South-Eastern Norway, 3184 Horten, Norway

**Keywords:** metal organic framework, 4,4′-biphenyl dicarboxylic acid, one-step hydrothermal method, tunable pore size, nanoplate structure, supercapacitors

## Abstract

Metal-organic frameworks (MOFs) have attracted significant research interest for supercapacitor applications due to their high-tunable conductivity and their structure’s pore size. In this work, we report a facile one-step hydrothermal method to synthesize nickel-based metal-organic frameworks (MOF) using organic linker 4,4′-biphenyl dicarboxylic acid (BPDC) for high-performance supercapacitors. The pore size of the Ni-BPDC-MOF nanostructure is tuned through different synthesization temperatures. Among them, the sample synthesized at 180 °C exhibits a nanoplate morphology with a specific surface area of 311.99 m^2^·g^−1^, a pore size distribution of 1–40 nm and an average diameter of ~29.2 nm. A high specific capacitance of 488 F·g^−1^ has been obtained at a current density of 1.0 A·g^−1^ in a 3 M KOH aqueous electrolyte. The electrode shows reliable cycling stability, with 85% retention after 2000 cycles. The hydrothermal process Ni-BPDC-MOF may provide a simple and efficient method to synthesize high-performance hybrid MOF composites for future electrochemical energy storage applications.

## 1. Introduction

The supercapacitor is a promising electrochemical energy storage device and plays an important role of bridging the gap between conventional capacitors and batteries [1,2]. Taking advantage of its long lifetime, high power density and fast charging-discharging rate, the supercapacitor attracts a lot of attention in fields ranging from portable electronic products to vehicle parts [3,4]. From the view of material design, the supercapacitive performance of electrode materials can be improved through a higher conductivity and surface area as well as an appropriate pore size for enhanced efficient ion transmission [5].

Compared with other types of electrode materials such as carbon-based materials, transition metal oxides and conducting polymers, the metal-organic framework (MOF), a porous crystalline material, is emerging as a promising material due to its variable structure, tunable pore size and high surface area [6]. Since the MOF is composed of central metal ions and organic ligands [7], it combines the high specific capacitance of pseudo capacitance and the reliable cycling stability of electric double-layer capacitance at the same time. So far, more than 20,000 different MOFs have been created, and the number is still growing [8]. Although they have relatively high porosity, few MOFs are used as electrode materials, mainly due to the insufficient pore size for the diffusion of electrolyte ions and the low conductivity [9].

Organic ligands play an important role in the morphology and electrical properties of MOF [10]. Among them, 4,4′-Biphenyl dicarboxylic acid (BPDC), 2, 6-Naphthalene dicarboxylic acid (NDC) and Benzene dicarboxylic acid (BDC) are the most common carboxylic acids used as the organic linkers for synthesizing MOF material. Some researchers have demonstrated that the molecular length of organic linkers influences the pore size and surface area of MOFs [11]. Longer linkers, such as BPDC, can provide MOFs with larger pores and surface areas and thus improve their capacitance properties [12,13]. Since the BPDC-based MOFs are inherently non-conductive and lack thermal and mechanical stability, a conscious method is to fabricate MOF-based composites with common transition metals such as Zn, Cd, Co, Mn, Cu and Ni [14,15,16,17], which may integrate the advantages and mitigate the drawbacks of individual components [16]. 

Herein, we attempt to design a BPDC-based nickel MOF (Ni-BPDC-MOF) with a different surface area, pore size and morphology for high-performance supercapacitor applications. Through a simple hydrothermal reaction between BPDC and nickel nitrate hexahydrate, the typical Ni-BPDC-MOF was prepared under four different hydrothermal temperatures to tune the pore size of its nanostructure. The morphology, surface area, porosity and conductivity of the Ni-BPDC-MOF varied with the hydrothermal temperature, leading to different capacitive properties. The sample fabricated at 180 °C showed a relatively high surface area and pore size and a good thermal stability, with a specific capacitance of 488 F·g^−1^ at 1.0 A·g^−1^ in the 3 M KOH aqueous electrolyte. Further, it exhibited excellent cycling stability, with a capacitance retention of 85% after 2000 cycles. 

## 2. Materials and Methods

### 2.1. Characterization Techniques and Electrochemical Measurements

The surface morphology of the Ni-BPDC-MOF was studied by field emission scanning electron microscopy (FESEM, SU8010, Hitachi Inc., Tokyo, Japan). Its crystal structure was tested by X-ray diffraction (XRD, D8 Advance, Bruker Inc., Billerica, MA, USA) in a 2θ range of 10–70 degrees with CuKα (α = 1.541 Å) radiation. The chemical analysis was evaluated under Fourier transform infrared spectroscopy (FTIR, NicoletiS10, Thermo Fisher Inc., Waltham, MA, USA) and X-ray photoelectron spectroscopy (XPS, EscaLab250Xi, Thermo Fisher Inc., Waltham, MA, USA). The thermal stability of the Ni-BPDC-MOF was investigated by thermo-gravimetric analysis (TGA, Pyris 1, PerkinElmer Inc., Waltham, MA, USA). To ensure the full decomposition, the sample was heated gradually up to 800 °C at a rate of ~10 °C/min in a nitrogen atmosphere. The Brunauer Emmet Teller (BET) surface area was characterized with the nitrogen adsorption method by a porosity analyzer (QuadraSorb S1, Quantachrome Inc., Graz, Austria).

The supercapacitive performance of the Ni-BPDC-MOF sample was studied by cyclic voltammetry (CV), electrochemical impedance spectra (EIS) and galvanostatic charge-discharge (GCD) tests employing an electrochemical workstation (IM6, Zahner-Elektrik GmbH Inc., Kronach Gundelsdorf, Germany). All of the tests were tested by using a standard three-electrode system, where the fabricated sample, platinum mesh and Hg/HgO were used as the working, counter and reference electrode, respectively. Then, 3.0 M KOH (85.0%, Sinopharm Chemical Reagent Co., Ltd., Shanghai, China) aqueous solution was served as the electrolyte in the electrochemical measurements due to the stability issue of the Ni-BPDC-MOF. For the EIS test, the perturbation potential was set as 5 mV, and the the frequency ranged from 0.01 Hz to 100 KHz.

### 2.2. Synthesis Methods

The Ni-BPDC-MOF was synthesized through a facile one-step hydrothermal method. In brief, 0.149 g of BPDC (97.0%, Sinopharm Chemical Reagent Co., Ltd., Shanghai, China) and 0.096 g of nickel nitrate hexahydrate (Ni(NO_3_)_2_·6H_2_O, 98.0%, Sinopharm Chemical Reagent Co., Ltd., Shanghai, China) were dissolved in 20 mL N, N-dimethylformamide (DMF, 99.5%, Sinopharm Chemical Reagent Co., Ltd., Shanghai, China) followed by continuous stirring. The molar ratio between the organic ligands and metal ions was kept as 3:2. Then, 0.032 g of sodium hydroxide (NaOH, 96.0%, Sinopharm Chemical Reagent Co., Ltd., Shanghai, China) was added into the solution. The configured solution was then shifted into a 50 mL Teflon-lined stainless steel autoclave. The hydrothermal process was done in atmosphere environment and kept at 180 °C for 10 h. Some light green precipitate appeared after the hydrothermal process, which was collected by centrifugation, washed in DMF and alcohol and tried in a nitrogen atmosphere. The temperature influence of the hydrothermal process was investigated with four different values: 120 °C, 150 °C, 180 °C and 210 °C, as described in the Supplementary Materials. The four types of Ni-BPDC-MOFs were activated at 120 °C in vacuum for 24 h [18].

For the consolidation of the sample on a substrate, nickel foam (Jiayisheng Electronics Co., Ltd., Kunshan, China) was adopted as the current collector, taking advantage of its three-dimensional structure for full electrolyte penetration, fast ion diffusion and excellent conductivity [19]. Then, 80 wt.% Ni-BPDC-MOF, 10 wt.% carbon black (Sinopharm Chemical Reagent Co., Ltd., Shanghai, China) and 10 wt.% polytetrafluoroethylene (60%, Aladdin Biochemical Technology Co., Ltd., Shanghai, China) were blended together in ethanol. The mixture was painted on one piece of nickel foam with a size of 10.0 × 20.0 mm, dried at 60 °C in a nitrogen atmosphere for 12 h and then compressed at a pressure of 8 MPa for the following electrochemical measurements.

## 3. Results and Discussion

**Figure 1:**
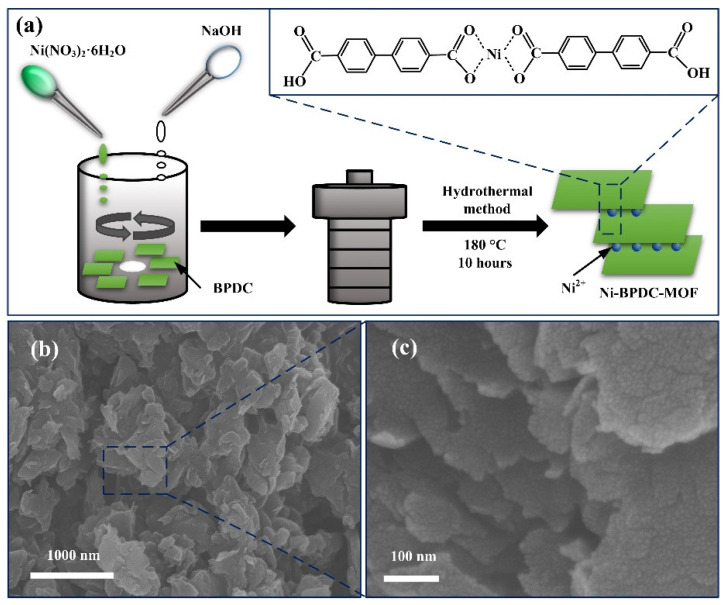
(**a**) Synthetic scheme for the preparation of Ni-BPDC-MOF nanoplates. (**b**,**c**) SEM graphs of the Ni-BPDC-MOF sample with different magnifications.

Figure 1 shows the typical morphology of the as-prepared Ni-BPDC-MOF. It can be seen that the Ni-BPDC-MOF is comprised of a lot of interconnected nanoplates with an approximate feature size of ~500 nm. As shown in Figure 1a, the high concentrated BPDC molecules (12.3 mmol/L) are easily ionized and construct a monolayer liquid membrane [17]. When added into the solution, the nickel ions are coordinated with the carboxyl ions on the top surface of one BPDC membrane and the bottom surface of another BPDC membrane simultaneously, forming the interconnected nanoplate morphology. The high magnification images of Figure 1b,c present a loose connection and large separation between two nanoplates [20], which may also allow for a continuous charge distribution as well as a large available surface to accelerate the electron transport throughout the framework. The SEM graphs by three different hydrothermal temperatures are displayed in Figure S1. With a lower temperature, the size of the nanoplate is small, with some cracks on its surface. The size increases with the increase in temperature. At 180 °C, the nanoplate is large and integrated. However, with higher temperatures, e.g., 210 °C, the structure seems to melt, leading to the smooth plate surface and edge.

**Figure 2:**
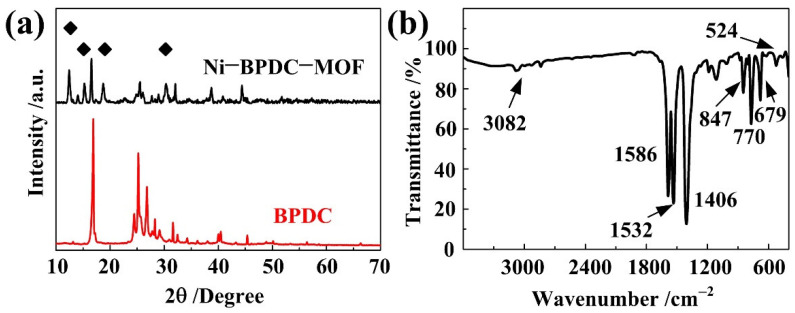
(**a**) XRD patterns of the Ni-BPDC-MOF and the pure BPDC samples. (**b**) FTIR spectrum of the Ni-BPDC-MOF sample.

Figure 2a shows the XRD patterns of the Ni-BPDC-MOF and the pure BPDC samples. Compared with those of the BPDC, several new diffraction peaks at 12.4°, 15.3°, 18.7° and 30.3° are observed in the case of the Ni-BPDC-MOF. The 2θ positions of these new peaks are similar to those of other reported isostructural Ni-BPDC-MOFs [21,22], suggesting a similar crystalline structure and the high crystallization of our samples. The XRD patterns of the Ni-BPDC-MOF with hydrothermal temperatures of 120 °C and 180 °C are compared in Figure S2. Since no more diffraction peaks appeared, the chemical structures of our samples may remain the same by changing the hydrothermal temperatures. The chemical bonds are further analyzed by FTIR. As shown in Figure 2b, a couple of intense bands at 847 cm^−1^, 1406 cm^−1^, 1532 cm^−1^ and 1586 cm^−1^ are observed, which could be assigned to the stretching vibration of the carboxylate groups (COO−) coordinated to the metal center [23]. Among them, the two bands located at 1406 cm^−1^ and 1586 cm^−1^ are assigned to the symmetric and asymmetric stretching modes of the COO− groups, respectively, which are in accordance with Rawool’s report [16]. The difference between these two bands indicates that the COO− groups are coordinated to the metal center through the bidentate mode. Three peaks at 679 cm^−1^, 770 cm^−1^ and 3082 cm^−1^ corresponded to the stretching vibration of the C−H bond, the ring deformation vibration of the benzene ring and the antiplane bending vibration of the aromatic ring in the BPDC, respectively [24]. The peak at 524 cm^−1^ was related to the Ni−O stretching vibration [21]. The XRD and FTIR analyses conclude a proper coordination between the organic ligand BPDC and the nickel ions. 

**Figure 3:**
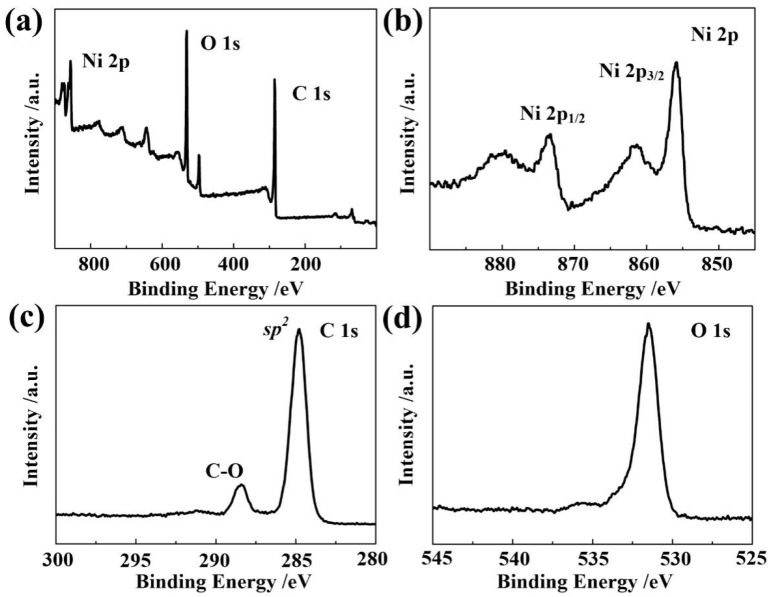
XPS spectrum of the Ni-BPDC-MOF sample. (**a**) Survey spectrum. (**b**–**d**) High resolution XPS spectrum of (**b**) Ni 2p, (**c**) C 1s and (**d**) O 1s.

The chemical valence states of the Ni-BPDC-MOF are studied by XPS. The survey spectrum in Figure 3a indicates the existence of C, O and Ni elements. Two major peaks located at 855.8 eV and 873.3 eV are observed in the spectra of Ni 2p (Figure 3b), which correspond to Ni 2p_1/2_ and Ni 2p_3/2_, respectively [25]. The other two peaks located at 861.3 eV and 879.6 eV are considered as satellite peaks for Ni^2+^ [26]. Figure 3c shows the C 1s of the Ni-BPDC-MOF. The two peaks at 284.8 eV and 288.6 eV represent the sp^2^ phase (C=C) in the benzene ring and (CO) bond, respectively [27]. The O 1s spectrum in Figure 3d exhibits one predominant peak at 531.5 eV, which is attributed to the adsorbed −OH on the surface of the Ni-BPDC-MOF [28], while another smaller peak at 536.5 eV is directly related to the hydrocarbon oxides in the BPDC [29].

**Figure 4:**
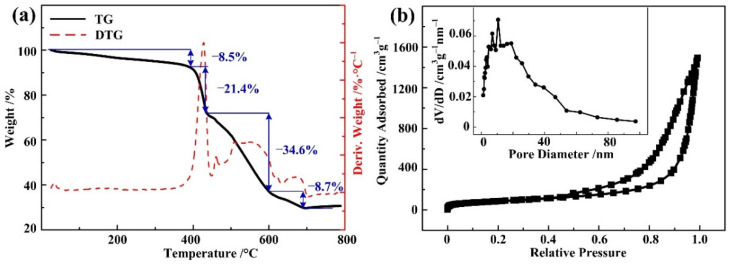
(**a**) TGA spectra of the Ni-BPDC-MOF sample. (**b**) N_2_ adsorption–desorption isotherms and the corresponding pore size distribution curve of the Ni-BPDC-MOF sample.

TGA is performed to evaluate the thermal stability of **the** Ni-BPDC-MOF. As shown in Figure 4a, four mass drops were observed. The first (8.5%) mass drop happened from the RT to 390 °C and was caused by the desorption of physiosorbed water and the chemisorbed organic solvent on the surface of the Ni-BPDC-MOF [30]. The second (21.4%) and the third (34.6%) mass drops happened sharply from 390 °C to 600 °C, with the fastest drop at 428 °C. This can be concluded as the decomposition of the Ni-BPDC-MOF’s organic skeleton and the generation of nickel oxide. The final mass loss was observed from 600 °C to 700 °C and is related to the exclusion of CO_2_ from the evolution of the carboxylic groups. When the applied temperature is higher than 700 °C, the mass remained stable, and less than 30% of the residual was left. The TGA demonstrates that the Ni-BPDC-MOF is thermally stable below 400 °C in the open air. 

Figure 4b shows the N_2_ adsorption–desorption isotherm and the corresponding pore size distribution curve of the Ni-BPDC-MOF. It shows a type-IV curve with a type-H3 hysteresis loop [31], indicating the highly porous structure of the as-prepared sample. The BET specific surface area is 311.99 m^2^·g^−1^. The pore sizes of the Ni-BPDC-MOF are further calculated by the Barrett−Joyner−Halenda (BJH) method. The pore size of the Ni-BPDC-MOF exhibits a bimodal distribution with a diameter of 1–40 nm, while the calculated average value is calculated as 29.2 nm. The average pore size seems to be higher than that of some common MOFs, which is proved by other BPDC-based MOFs with similar structures [20,32]. The relatively larger surface area and pore size may be due to the micropores produced by the long organic linker BPDC and the mesopores caused by the stacked nanoplates. The relatively larger surface area and pore size are likely to lead to the easy diffusion of electrolyte ions, which is beneficial to high charge storage capacity [16]. 

The adsorption–desorption isotherms and pore size distribution curves of the other samples are shown in Figure S4. It can be seen that the sample synthesized at 180 °C has the largest surface area. The result is in good agreement with the SEM observation. With lower temperatures, the size of the nanoplate is too small and cannot supply enough surface area, while with higher temperatures, the nanoplate is melted and sticks with the other, so the surface area value drops again. A temperature of 180 °C is considered the optimized value for Ni-BPDC-MOF synthesization. 

**Figure 5:**
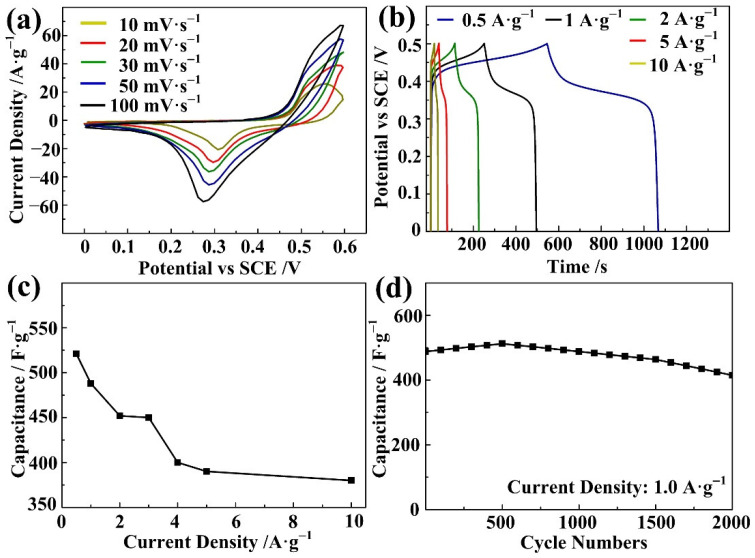
Capacitance performances of the Ni-BPDC-MOF sample. (**a**) CV curves at different scan rates. (**b**) GCD curves at different current densities. (**c**) Specific capacitances at different current densities. (**d**) Cyclic stability.

Electrochemical measurements have been carried out to evaluate the supercapacitive performance of the Ni-BPDC-MOF. Figure 5a depicts the CV curves of the Ni-BPDC-MOF sample at three different scan rates from 10 to 100 mV·s^−1^, with a potential range 0–0.6 V. All of the CV curves displayed a couple of obvious redox peaks, indicating the typical pseudocapacitive property of the samples [33]. With increasing scan rates, the oxidation peak is shifted positively, while the reduction peak is shifted negatively, resulting from the resistance of the electrodes [34]. The pseudocapacitance is generated from faradic redox reactions of the intercalation and deintercalation of the OH^−^ during electrochemical reactions in the Ni-BPDC-MOF. The anodic and cathodic peaks in the CV curves are considered to be different oxidation states of nickel, corresponding to the following process:

(1) $$\mathrm{Ni}{\left(\mathrm{II}\right)}_{s}+{\mathrm{OH}}^{-}↔\mathrm{Ni}\left(\mathrm{II}\right){\left(\mathrm{OH}\right)}_{\mathrm{ad}}+e^{-}$$

(2) $$\mathrm{Ni}\left(\mathrm{II}\right){\left(\mathrm{OH}\right)}_{\mathrm{ad}}↔\mathrm{Ni}\left(\mathrm{III}\right){\left(\mathrm{OH}\right)}_{\mathrm{ad}}+e^{-}$$

Figure 5b presents the GCD curves of the Ni-BPDC-MOF with three different current densities. The charge time and discharge time in each GCD curve are almost equal, demonstrating a good capacitance performance and the reversible redox process of the Ni-BPDC-MOF sample. For the discharging part, an obvious potential plateau at 0.43 V is observed. This plateau is interpreted as the electrochemical adsorption–desorption and the faradic redox reaction process on the interface of the electrode and electrolyte [35], which is in accordance with the CV curves. The specific capacitance is calculated from the discharging time of the GCD curve. At the commonly used current density (1.0 A·g^−1^), the Ni-BPDC-MOF sample delivers a value of 488 F·g^−1^. Figure 5c concludes the specific capacitances of the Ni-BPDC-MOF with different current densities. With increasing current densities, the calculated capacitance decreases. However, even the current density is changed from 0.5 F·g^−1^ to 10 F·g^−1^. The capacitance is dropped from 521 F·g^−1^ to 380 F·g^−1^, with 72.9% of the capacitance retained. The result shows the good rate capability of the Ni-BPDC-MOF. The GCD test was circulated 2000 times at a current density of 1.0 A·g^−1^ to evaluate its cyclic stability. As shown in Figure 5d, more than 85.0% of the specific capacitance is reserved. In the first 500 cycles of the test, the capacitance increased, which may result from the electro-activation of the active material [36]. The value was decreased from 500 to 2000 cycles continuously, owing to the shedding of active material from the nickel foam during the cycling test.

**Figure 6:**
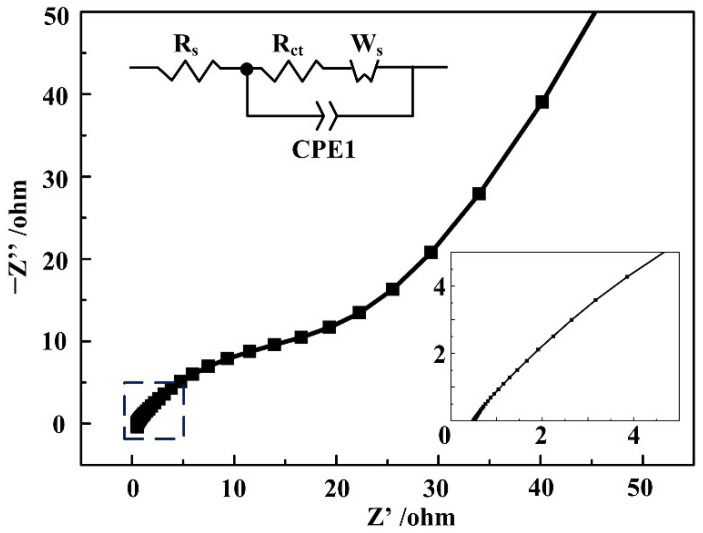
Nyquist plots of the Ni-BPDC-MOF sample.

In Figure 6, *R_s_* is the internal resistance of the electrode, including the contact resistance of the active material and substrate, the intrinsic resistance of the active material and the ionic resistance of the electrolyte. *R_ct_* and *W_s_* are the charge transfer resistance and Warburg diffusion impedance, which are related to the pseudocapacitive reaction. CPE1 is the double-layer capacitance element and can be neglected in this research. In the low frequency part, the slope of the straight lines in the low frequency is larger than 45°, confirming a smaller *W_s_* and a fast migration of the ions [37]. *R_ct_* and *R_s_* are calculated from the radius of the semicircle and the intercept of the real axis in the high frequency range, with relatively low values of 0.49 Ω and 18.55 Ω [38], respectively. The EIS result indicates that the Ni-BPDC-MOF has a good ability to deliver charges on the interface of the electrode and electrolyte and thus led to a positive effect on its specific capacitance. The relationship between the hydrothermal temperature and the supercapacitive performance of the Ni-BPDC-MOF is displayed in Figure S4 and Table S1. All of the Ni-BPDC-MOFs exhibit a typical seudocapacitive property, and the sample synthesized at 180 °C had the highest capacitance.

Table 1 compares recent reports on the porosity and supercapacitive performance of BPDC-based MOF materials. The specific capacitance is superior to that found by most other researchers in the absence of the highest surface area value. For the research on BPDC-based porous carbon nanoparticles (C-BPDC) [15,30], the specific surface area is even 2~3 times higher than that found in our report. However, because of their small pore size, the electrode materials may be not well-soaked in the electrolyte, leading to a relatively low specific capacitance. For the BPDC-based metal nanoparticles (Al-BPDC, Ni-BPDC) [16,17,39], the surface area and pore size may reach a sufficient amount for the better intercalation of electrolyte ions. Still, our report has the lowest *R_ct_* value, which might be related to our unique structure: the interconnected network of the Ni-BPDC nanoplates results in a continuous charge distribution, leading to a faster electron transport throughout the whole framework and a lower *R_ct_* value [16].

## 4. Conclusions

In conclusion, we demonstrate a facile one-step hydrothermal method for synthesizing a BPDC-based nickel MOF with an optimized reaction temperature of 180 °C. A chemical characterization confirmed a good coordination between the organic ligand BPDC and the nickel ions. The as-synthesized Ni-BPDC-MOF presents a nanoplate structure with a relatively high specific surface area of 311.99 m^2^·g^−1^, a pore size distribution of 1–20 nm and an average diameter of 29.2 nm. As a result, the capacitance properties of the Ni-BPDC-MOF exhibited a great performance, with a specific capacitance of 488 F·g^−1^ at a current density of 1.0 A·g^−1^. The capacitance retention is kept at 85% of its initial value after 2000 cycles. This study demonstrates a simple synthesizing method for high-performance BPDC-based MOF material, which may become a promising candidate for electrochemical capacitive energy storage.

## Figures and Tables

**Table 1:** Comparison of the present work with previously reported BPDC-based MOFs for porosity and supercapacitive performance.

Sample	Surface Area (m^2^·g^−1^)	Average Diameter (nm)	Electrolyte	Scan Rate (mV·s^−1^)	Current Density (A·g^−1^)	*R_ct_* (Ω)	Capacitance (F·g^−1^)	Ref.
C-BPDC	1137	4.2	6M KOH	1	—	0.34	170	[40]
Al-BPDC	415.2	18.5	6M KOH	—	0.25	3.25	119	[39]
C-BPDC	843	0.53	6M KOH	—	1	1.23	256	[41]
Ni-BPDC	347	32.7	6M KOH	—	1	1.10	328	[16]
Ni-BPDC	—	—	6M KOH	—	1	0.65	432	[17]
**Ni-BPDC**	**311.99**	**29.16**	**3M KOH**	**—**	**1**	**0.49**	**488**	**This Work**

## Data Availability

The data presented in this study are available on request from the corresponding author.

## Supplementary Materials

The following supporting information can be downloaded at: https://www.mdpi.com/article/10.3390/nano12122062/s1, Figure S1: SEM graphs of Ni-BPDC-MOF samples with different hydrothermal temperature; Figure S2: XRD patterns of Ni-BPDC-MOF with hydrothermal temperatures of 120 °C and 180 °C; Figure S3: N2 adsorption/desorption isotherms and corresponding pore size distribution curve of Ni-BPDC-MOF samples with different hydrothermal temperature; Figure S4: Capacitance performances of Ni-BPDC-MOF samples with different hydrothermal temperature; Table S1: The comparison for porosity and supercapacitive performance of Ni-BPDC-MOF samples with different hydrothermal temperature.
