# Supplementary material for: Facile Synthesis of 4,4′-biphenyl Dicarboxylic Acid-Based Nickel Metal Organic Frameworks with a Tunable Pore Size towards High-Performance Supercapacitors
Source: Nanomaterials (Basel). 2022 Jun 15;12(12):2062. doi: 10.3390/nano12122062 (PMC9227198; doi:10.3390/nano12122062)
Supplement: Supplementary file 1 [file nanomaterials-12-02062-s001.zip › nanomaterials-1735766-supplementary.pdf]

nanomaterials

Supplementary Material

Facile Synthesis of 4, 4'-biphenyl Dicarboxylic Acid-Based Nickel Metal Organic Frameworks with a Tunable Pore Size towards High-Performance Supercapacitors

Wenlei Zhang [1], Hongwei Yin [1], Zhichao Yu [1], Xiaoxia Jia [1], Jianguo Liang [2], Gang Li [1,3,*], Yan Li [2] and Kaiying Wang [1,4,*]

[1] Institute of Energy Innovation, College of Materials Science and Engineering & College of Information and Computer, Taiyuan University of Technology, Taiyuan 030024, China; zhangwenlei@tyut.edu.cn (W.Z.); yinhongwei@tyut.edu.cn (H.Y.); yuzhichao@tyut.edu.cn (Z.Y.); jiaxiaoxia@tyut.edu.cn (X.J.)
[2] College of Mechanical and Vehicle Engineering, Taiyuan University of Technology, Taiyuan 030024, China; liangjianguo20@tyut.edu.cn
[3] College of Physics and Information Engineering, Minnan Normal University, Zhangzhou 361000, China; liyan_nmsd@163.com
[4] Department of Microsystems-IMS, University of South-Eastern Norway, 3184 Horten, Norway
* Correspondence: ligang02@tyut.edu.cn (G.L.); kaiying.wang@usn.no (K.W.)

Citation: Zhang, W.; Yin, H.; Yu, Z.; Jia, X.; Liang, J.; Li, G.; Li, Y. Facile Synthesis of 4, 4′-biphenyl Dicarboxylic Acid-Based Nickel Metal Organic Frameworks with a Tunable Pore Size towards High-Performance Supercapacitors. *Nanomaterials* **2022**, *12*, 2062. https://doi.org/10.3390/nano12122062

Academic Editor: Jung Woo Lee

Received: 5 May 2022
Accepted: 13 June 2022
Published: 15 June 2022

Publisher's Note: MDPI stays neu-tral with regard to jurisdictional claims in published maps and institu-tional affiliations.

Copyright: © 2022 by the authors. Submitted for possible open access publication under the terms and conditions of the Creative Commons Attribution (CC BY) license (https://creativecommons.org/licenses/by/4.0/).

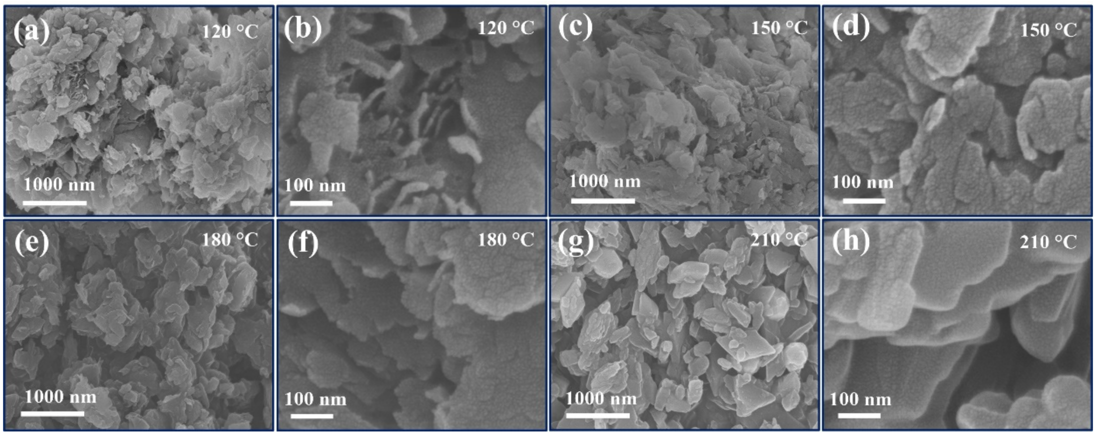

Figure S1. SEM graphs of Ni-BPDC-MOF samples with different hydrothermal temperature. (**a**,**b**) Sample synthetized at 120 °C. (**c**,**d**) Sample synthetized at 150 °C. (**e**,**f**) Sample synthetized at 180 °C. (**g**,**h**) Sample synthetized at 210 °C.

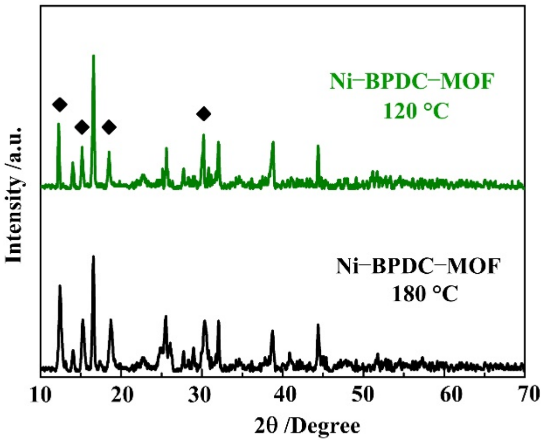

Figure S2. XRD patterns of Ni−BPDC−MOF with hydrothermal temperatures of 120 °C and 180 °C. The characteristic diffraction peaks of Ni−BPDC−MOF are remarked in diamond pattern.

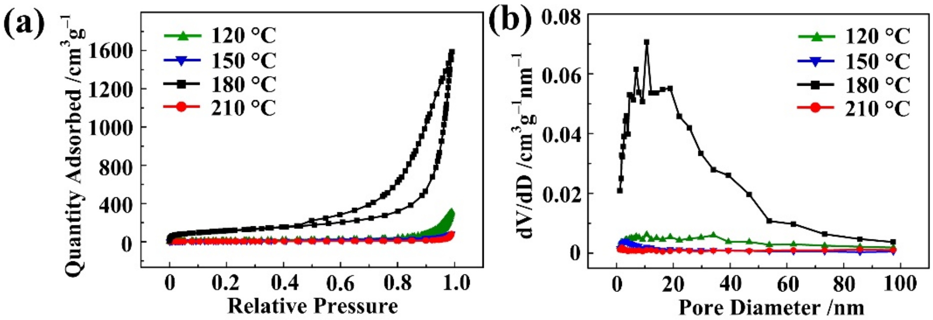

Figure S3. (**a**) N_2 adsorption/desorption isotherms and (**b**) corresponding pore size distribution curve of Ni-BPDC-MOF samples with different hydrothermal temperature.

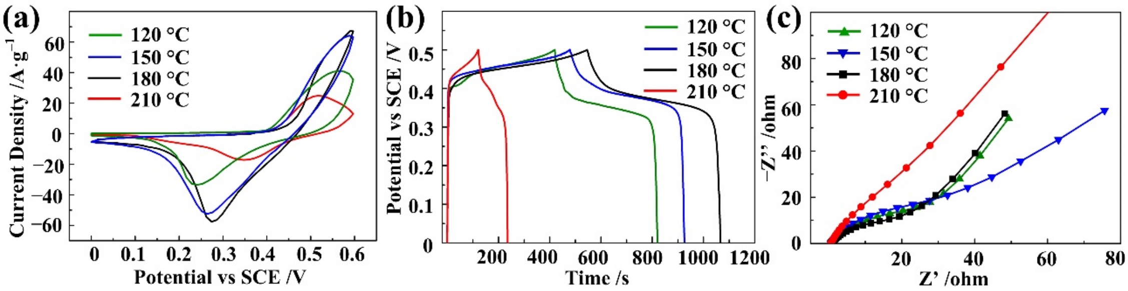

Figure S4. Capacitance performances of Ni-BPDC-MOF samples with different hydrothermal temperature. (**a**) CV curves at scan rate of 100 $mV \cdot s^{-1}$. (**b**) GCD curves at current density of 0.5 $A \cdot g^{-1}$. (**c**) Nyquist plots.

Table S1. The comparison for porosity and supercapacitive performance of Ni-BPDC-MOF samples with different hydrothermal temperature. The capacitance was calculated from GCD curves with current density of 0.5 $F \cdot g^{-1}$.

Synthetized Temperature	Surface Area ($m^2 \cdot g^{-1}$)	Average Diameter (nm)	R_{ct} (Ω)	R_s (Ω)	Capacitance ($F \cdot g^{-1}$)
120 °C	137.72	12.54	21.43	0.52	400
150 °C	87.10	8.69	21.08	0.61	448
**180 °C**	**311.99**	**29.16**	**18.55**	**0.49**	**521**
210 °C	62.00	7.54	36.22	0.45	116
